# Development and Characterization of a Low-Cost Sensors System for an Acoustic Test Bench

**DOI:** 10.3390/s20226663

**Published:** 2020-11-20

**Authors:** Ciro Moreno-Ramírez, Carmen Iniesta, Alejandro González, José Luis Olazagoitia

**Affiliations:** Industrial Engineering and Automotive Department, Universidad Antonio de Nebrija, Pirineos 55, 28040 Madrid, Spain; cmorenora@nebrija.es (C.M.-R.); miniesta@nebrija.es (C.I.); agonzalezmu@nebrija.es (A.G.)

**Keywords:** DAQ, data acquisition system, low cost, reliability, Arduino, pressure sensing, acoustics, DeltaEC

## Abstract

Existing acoustic test benches are usually costly devices based on proprietary designs, sensors, and acquisition devices. In this paper, a low-cost test bench for acoustic purposes is introduced. The design of the test bench takes into account not only the low-cost mechanical design, but also uses low-cost sensors and control boards. This test bench has been designed for a range of signals compatible with those used by thermoacoustic engines, but it can be useful for applications with similar requirements. Taking advantage of an auxiliary pressure reference, low-cost unidirectional differential pressure sensors can be used to significantly increase the accuracy of the sampling system. The acoustic and mechanical design and development are presented along with the sampling system and the sensors arrangement implemented. Both the sensor and sampling system are evaluated by comparison with a high-fidelity sound acquisition system. An unexpected effect on the time error values distribution of the low-cost acquisition system is found and described. Finally, the errors introduced by the system and the sensors in terms of time and pressure sampling are characterized. As a result, the low-cost system’s accuracy has been satisfactory assessed and validated for the conditions expected in thermoacoustic experiments in terms of frequency and dynamic pressure.

## 1. Introduction

Development platforms based on low-cost control systems (e.g., Arduino) are becoming increasingly popular. These systems allow people with limited knowledge or economic capacity to test concepts in a simple way and at a limited cost. In this sense, applications related to the Internet of Things (IoT), usually associated with this type of platform, are becoming increasingly popular.

In the relevant literature, it is possible to find in recent years many applications related to data acquisition, monitoring and analysis with low-cost systems. For example, an Arduino-based five-channel acquisition system for use in an aerodynamics laboratory is presented in [1]. A system for remote monitoring of temperature and humidity with an Arduino Uno was presented in [2]. Continuous conductivity monitoring in marine environments was presented in [3] using an Arduino Mega2560 and an Arduino UNO. An Arduino Nano was used as a data acquisition system for a gas-discharge plasma diagnostics [4]. Industrial acquisition and control systems have also been developed as in [5] using an Arduino Due. It is also possible to find its application in high performance systems in data acquisition in vehicle applications, as in [6] where the limits in terms of frequency acquisition are explored and its performance is compared with professional acquisition systems, showing great performance. This paper also makes a comparison (in time and frequency) of the performance of low-cost accelerometers against professional sensors with sinusoidal displacement signals. The robustness of these Arduino based systems was studied in [7] concluding that it is possible to use them in high frequency systems and real time applications by implementing additional security layers to ensure safe and reliable data transmission at a high acquisition rate.

As the above examples show, the range of applications where acquisition systems based on these low-cost platforms have been implemented is innumerable. It is possible to assure that, to a certain extent, these low-cost electronic systems can become, in performance and reliability in some applications, a technologically viable alternative and at a very competitive price, with respect to high-priced dedicated systems. This paper will use a low-cost acquisition system (based on Arduino) with low-cost sensors, which will be compared with professional sensors to validate their accuracy and functionality. Based on the results obtained in previous studies [6] with accelerometers in Arduino and its robustness [7], it is expected that the developed system will be equally functional in the determination of the variable acoustic field in thermoacoustic systems.

Thermoacoustic engines have been studied for over three decades [8], and because of their simplicity and theoretical efficiency, they now represent credible alternatives to other types of thermodynamic engines for applications focused on the processes of waste heat recovery. For example, in [9] the application of thermoacoustic Stirling engines is proposed as a simple, reliable, and efficient solution to improve the overall energy efficiency of the LNG power generation system. Another waste heat recovery application can also be found in [10], oriented to improve the overall energy efficiency of the vehicle using thermoacoustic technology to recover wasted heat and convert part of it to electric power. Although much has been done in terms of the development of these engines and the basic understanding of their operation, efforts are still needed to promote their applications. Among the key points that still require further research are the precise characterization of sound propagation and distribution of the acoustic field by properly quantifying acoustic power distribution [11] and non-linear effects such as acoustic streaming [12] or the generation of higher harmonics [13].

The acoustic power flowing along a duct of area A can be determined from accurate measurements of the pressure amplitude and phase at two transducers 1 and 2 that are separated by a short distance ∆x along the duct by the two-microphone method [14], widely used in thermoacoustics. Unfortunately, by now, those effects cause significant error to the result. Hence, a lock-in amplifier (or other expensive instrumentation) is used due to its high harmonic-suppression facility, for successful measurements at high levels of accuracy (i.e., 2% in pressure phasor) [15,16].

This article seeks to quantify the levels of accuracy that one may achieve for dynamic pressure measurements under controlled laboratory conditions for which the measured dynamic pressure by a low-cost acoustic test is expected to compare well with theoretical predictions. This article presents the design and development of a low-cost acoustic test bench designed primarily for the signal characterization of thermoacoustic systems, but which may be applicable to other acoustic applications. Some low-cost acoustic test stands for different types of applications can be found in the literature. However, none have been found applied to thermoacoustic systems. For example, in [17] a low-cost acoustic probe is developed for the estimation of the velocity of wave pulses. In [18] a study of the state of the art for vibroacoustic conditioning of internal combustion engines was carried out. In [19], an extension of a test bench for acoustic measurements for turbochargers was developed. More teaching-oriented applications can also be found, as in [20], but oriented to the implementation of a test bench for noise reduction.

On the other hand, apart from thermoacoustic applications, it is possible to find an extensive research to the measurement of acoustic power, but by means far from the low cost. For example, the method of using two adjacent pressure microphones has been widely used since the 1940s [21]. This method is based on the fact that the sound intensity can be determined from the signals acquired by two adjacent pressure sensors. In this case the average of the two signals is used to calculate the pressure and its difference to determine its speed. Recently [22], an experimental validation has been presented using a laser vibrometer to determine sound power using acoustic radiation modes. In this case, apart from calculating the sound power from the acoustic radiation modes, a novel experimental validation and comparison with other sound power measurement standards over a wide frequency range using an expensive laser vibrometer is provided. Another paper [23] presents a method of measuring the responses to a non-stationary sound impulse using professional acoustic equipment and identifying the signals by means of a recursive least-squares algorithm. Acoustic measurement in the design of internal combustion machines is a typical application for improving their performance [24]. The chillers in these systems impose a longitudinal thermal gradient on the air enclosed in narrow channels. In this case a measurement-calibration system is used to identify the causes of the resonance. In addition, it is indicated that a semi-infinite pipe is used due to the influence that the microphones used have on the results. In addition, possible errors have been identified in the measurement of sound pressure in long-duct pipes [25] when the measuring points are at certain distances from the sound sources.

This paper focuses on the design, development, and validation of a low-cost test bench, both in its design and its instrumentation, for the acquisition and identification of acoustic signals in thermo-acoustic applications. For this reason, works presented in the state of the art related to (1) acquisition and low-cost sensors, (2) acquisition systems used in thermoacoustics, and (3) acoustic signal acquisition systems have been presented. Regarding data acquisition through low-cost systems, we have found papers that use Arduino-type platforms, which allow the acquisition of data at high frequency. These requirements are similar to those needed in the application presented in this paper. On the other hand, it has been seen that there are acquisition systems integrated into thermo-acoustic systems, but to ensure the accuracy of the measurements they need expensive instruments, so they are out of the scope of this paper, focused on finding a low-cost solution. On the other hand, with respect to the published systems related to the acquisition of acoustic field data, different techniques are used, the most common one based on the separation of two microphones at a given distance, in addition to advanced instruments also of high cost.

In short, after reviewing the state of the art, we can conclude that there are several techniques and methods that allow us to measure the characteristics of an acoustic wave. However, the methods used for this purpose require expensive laboratory equipment. Moreover, despite the great boom in the use of low-cost systems and platforms in different types of applications, there are few cases in which they are used for acoustic studies. The characteristics of systems with modern thermoacoustic technology impose additional restrictions and requirements to be able to measure the power of the acoustic wave that travels inside them.

The novelty of this article focuses on the assessment and validation of a low-cost acquisition and identification system in an acoustic test bench for acoustic signals in thermoacoustic applications. The system is characterized using low-cost technology both for the creation of the bench (using 3D printing), and for the monitoring system (low-cost absolute and differential pressure sensors) and data acquisition (using Arduino based systems). Despite the low cost, the system achieves high acquisition accuracy thanks to the implementation of an auxiliary reference pressure line for the sensors. In addition, the use of this low-cost technology is compared with professional sensors and acquisition systems to determine the extent to which this approach is feasible. The article makes low-cost benchmark acquisitions at different frequencies and provides recommendations for achieving reliable and robust measurements, as well as determining the uncertainties and limitations of these systems.

Although the final application of the development of the acoustic bench presented here is thermoacoustics, the development carried out does not restrict its general usefulness. The low-cost techniques of bench design, sensorization and data acquisition can be directly applied to other acoustic fields. Therefore, this development is expected to be of general interest when acoustic characteristics, sound power or acoustic impedance in ducts are to be measured, or when low-cost pressure sensors with Arduino type acquisition systems are to be used in other applications.

This article is distributed as follows: Section 2 presents the design and development of the acoustic test bench. Section 3 presents the low-cost acquisition systems and their comparison with professional systems. Section 4 discusses the results and presents the conclusions.

## 2. Acoustic Test Bench

### 2.1. Design of DeltaEC Model

The software DeltaEC (Design Environment for Low-amplitude ThermoAcoustic Energy Conversion) developed by Los Alamos National Laboratory [26] currently represents the most widely used and effective way to design and analyze thermoacoustic systems in one dimension. DeltaEC is a very helpful tool, which provides the necessary knowledge of the acoustic field characteristics under the assumptions of linear acoustic approach: plane waves, laminar flow, and no streaming. In order to design the loudspeaker-resonator assembly, a numerical DeltaEC code has been build. According to low-order linear approximation [27], this software integrates the three-dimensional phenomena in the thermoacoustic systems with the one-dimensional governing wave propagation equations:

(1) $$\frac{dp_{1}}{d_{x}}=-\frac{i\omega \rho_{m}}{A\left(1-f_{v}\right)}U_{1}$$

(2) $$\frac{dU_{1}}{d_{x}}=-\frac{i\omega A\left[1+\left(\gamma -1\right)f_{k}\right]}{\gamma p_{m}}p_{1}+\frac{\left(f_{k}-f_{v}\right)}{\left(1-f_{v}\right)\left(1-\sigma \right)T_{m}}\frac{dT_{m}}{dx}U_{1}$$

where $p_{1}$ and $U_{1}$ are the pressure wave amplitude and the volume flow rate, both are time-dependent variables, oscillating along the *x*-axis. ω, $\rho_{m}$, $p_{m}$, A, γ are angular frequency, mean density, mean pressure cross-sectional area and specific heat ratio, respectively. $\;f_{v},\;f_{k}$ are the Rott’s thermoviscous functions. These time-dependent variables, oscillating along *x*-axis and expressed in phasor notation are:

(3) $$p\left(x,t\right)=p_{m}+Re\left[p_{1}\left(x\right)e^{i\omega t}\right]$$

(4) $$U\left(x,t\right)=Re\left[U_{1}\left(x\right)e^{i\omega t}\right]$$

For acoustic networks, the inertance l and compliance c, describes respectively, the inertial properties and the elastic properties of the gas for a channel with length Δx:

(5) $$l=\frac{\rho_{m}}{A}\frac{1-Re\left[f_{v}\right]}{{\left|1-f_{v}\right|}^{2}}$$

(6) $$c=\frac{A}{\gamma p_{m}}\left(1+\left[\gamma -1\right]Re\left[f_{k}\right]\right)$$

Additionally, the effect of viscosity on oscillating gas velocity and the effect of the gas thermal contact with the solid surface on oscillating temperature, adds, respectively, viscous resistance $r_{v}$ in series with inertance and thermal-relaxation resistance $\;r_{k\;}$ in parallel with compliance:

(7) $$r_{v}=\frac{\omega \rho_{m}}{A}\frac{Im\left[-f_{v}\right]}{{\left|1-f_{v}\right|}^{2}}$$

(8) $$\frac{1}{r_{k}}=\frac{\gamma -1}{\gamma }\frac{\omega AIm\left[-f_{k}\right]}{p_{m}}$$

Combining Equations (5) and (7), and Equations (6) and (8), the governing wave propagation equations into the acoustic network provide a complete description of the dynamics linking $\;p_{1}\;$ and $\;U_{1}$ for acoustic networks and can be rewritten in the form:

(9) $$dp_{1}=-\left(i\omega ldx+r_{v}dx\right)U_{1}$$

(10) $$dU_{1}=-\left(i\omega cdx+\frac{1}{r_{k}}dx\right)p_{1}+\frac{\left(f_{k}-f_{v}\right)dT_{m}}{\left(1-f_{v}\right)\left(1-\sigma \right)T_{m}}U_{1}$$

The DeltaEC design tool has been adopted to define the acoustic design of the loudspeaker-resonator assembly. The model allows to obtain the design parameters of the acoustic bench by matching the acoustic resonance frequency of the resonator with the mechanical resonance frequency of the speaker at 60 Hz. In this way the number of harmonics generated is irrelevant, so no distortions are produced. Therefore, the system remains harmonic and the linear analysis on which the design is based is fulfilled with respect to the acoustic propagation. This is achieved by modifying the elastic constant of the electrodynamic speaker by varying the volume of its rear cavity.

### 2.2. Mechanical Design

The DeltaEC code built is an approximation of the real device mainly due to the software inability to model the non-linear effects generated at high amplitudes, but the use of this software is that it has given useful results during thermoacoustic device designs. Therefore, the experimental setup is based on the linear DeltaEC model to generate an oscillating flow for the acoustic frequency (i.e., >20 Hz). An electromechanical system (i.e., acoustic loudspeakers) is used to generate an oscillating flow at the resonance frequency of the system to achieve measurable pressure amplitudes.

**Figure 1:**
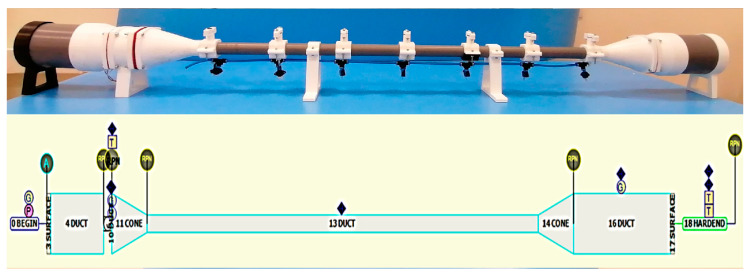
Acoustic test bench design. Above is a photograph of the acoustic bench built based on the DeltaEC model, the scheme of which is illustrated below.

The resulting mechanical configuration of the model for the acoustic test bench employed is shown in Figure 1. It consists of a loudspeaker-resonator assembly that is divided into three perfectly coupled watertight sections. The central section is a 1107-m long cylindrical PVC pipe with an internal diameter of 32 mm that acts as a resonator. It has seven locations spaced by 15.8 cm, where holes have been drilled that allow several types of sensors to be mounted in parallel to simultaneously measure the dynamic pressure, or that can be sealed when not in use. The sections located at the ends of the system are acoustic cavities in the form of a cylindrical PVC tube with an internal diameter of 110 mm and a length of 15 cm where the speakers are housed. The resonator is attached to the speaker cavities by means of conical pieces made ad-hoc with additive manufacturing in PLA. Its dimensions, also provided by the model, ensure a smooth transition of the cross-section, thus complying with the assumptions of linear theory, as far as possible. The mechanical design of the bench allows several configurations to be combined for testing: open and closed mode, with one or two speakers. Unpressurized air is used throughout this study. From the model, a single electrodynamic loudspeaker (Monacor SP-60/8) was chosen as the acoustic wave source in the system, which is closed at the other end by a rigid surface using a 27 cm conical resonant cavity that, when not housing a second loudspeaker, also forms part of the central resonator, avoiding the effect of non-linear propagation. [8].

**Figure 2:**
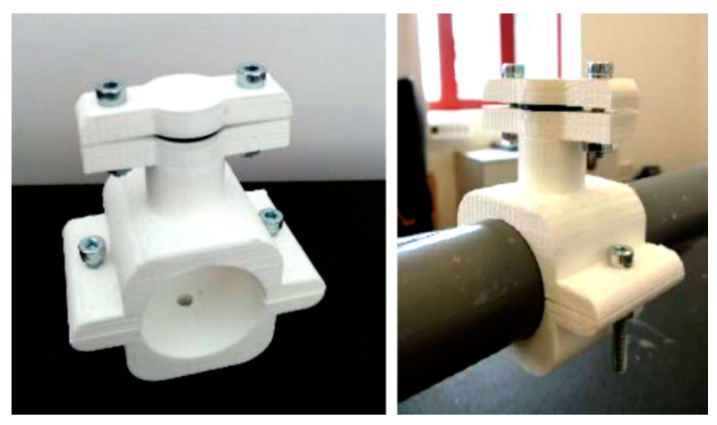
Clamp with stopper (**left**), mounted on the bench (**right**).

Figure 2 shows the coupling clamp made of additive PLA manufacture for mounting the sensors to the acoustic bench. These components are carefully designed so that no leakage occurs in the system and that they act properly as a support for two different types of sensors for simultaneous pressure measurements, with the same time base.

### 2.3. Acquisition System

**Figure 3:**
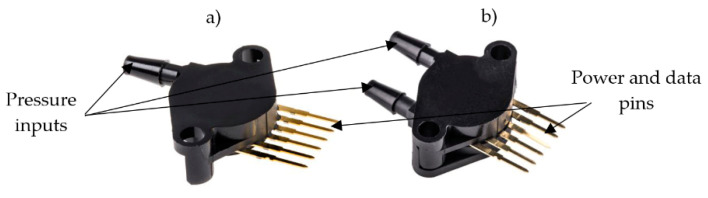
Low-cost pressure sensors used in the acoustic bench. (**a**) MPX5100AP sensor; (**b**) MPX5050DP sensor.

To measure and analyze the pressures produced in the acoustic bench, a low-cost data acquisition system (LC-DAQ) is proposed. The DAQ must be capable of measuring analog signals at least twice the frequency of the wave emitted by the driver. In addition, the system must maintain a low price, easy operability and optimal accuracy for the acoustic application. In order to choose a suitable acquisition platform, existing low-cost pressure sensors must be analyzed. The two selected sensors that meet the requirements are the MPX5100AP absolute pressure sensor and the MPX5050DP differential pressure sensor. Using differential sensors versus absolute sensors has two advantages. On the one hand, the increase in measurement accuracy due to the reduction of the full scale. On the other hand, the reduction of the reference voltage of the ADC to increase the resolution. The MPX5100AP sensor is a piezoresistive transducer specially designed for applications where samples are taken by a microcontroller with analog inputs. This sensor has been designed to measure the absolute air pressure inside thermal engines and has a sampling range from 15 to 115 kPa. The analog communication of the transducer has a range that varies between 0.2 and 4.9 V depending on the acquired pressure. The sensitivity is 45 mV/kPa. On the other hand, the MPX5050DP differential pressure sensor is a piezoresistive transducer designed for different applications. As in the previous case, the differential sensor is specially designed to be used together with a microcontroller. It has a measurement range from 0 to 50 kPa and an output signal from 0.2 to 4.7 V and its sensitivity is 90 mV/kPa, double of the absolute pressure sensor. The sensor has two physical inputs, one for the reference pressure and the other for the measured pressure. Figure 3 shows the two sensors used, where Figure 3a represents the MPX5100AP sensor and Figure 3b the MPX5050DP sensor.

**Figure 4:**
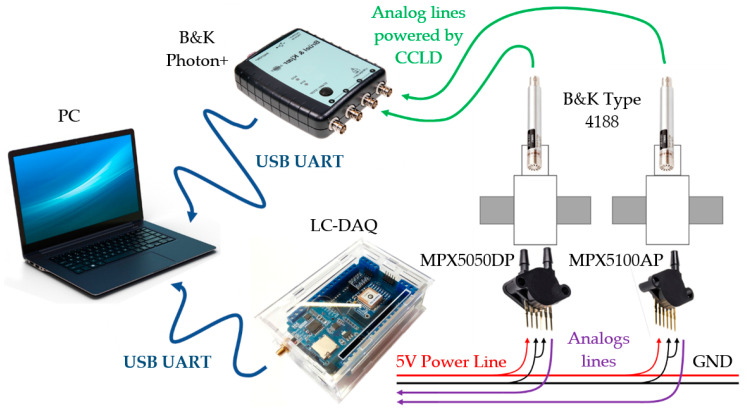
Wiring diagram.

The information sent by the sensors in analog form is collected by the LC-DAQ system. This acquisition system consists of an Arduino Mega development board together with a low-cost PC. Arduino Mega is a development board based on the ATmega2560 microcontroller. The microcontroller has a 16-channel analog with a 10-bit A/D converter and a crystal oscillator with a frequency of 16 MHz. The board has 16 analog inputs with an input range from 0 to 5 V. The voltage reference can be selected by software in 1.1 V or 5 V to change the resolution of the measurement. This range is optimal for the sensors used. Each one of the channels has 10 bits of resolution with a measurement’s range of 4.88 mV. The Arduino board samples the analog inputs in each cycle of the firmware and sends them via serial communication over USB by means of a byte chain to the microcomputer. The protocol for sending data by serial communication was developed in [7]. The PC saves the data collected during the acquisition in a csv file which is post-processed. Figure 4 shows the data communication from the LC-DAQ, with a Raspberry Pi 3 B+ as a PC.

To compare the accuracy of the data obtained by the LC-DAQ, a professional acquisition system is used together with calibrated professional sensors. In this case, the Brüel and Kjær company’s Photon+ acquisition system was used. Photon+ is a professional plug-and-play (PnP) system that allows high-precision measurements thanks to its four analog inputs with 24-bit resolution. The voltage input ranges can be varied by increasing the measurement accuracy. In the maximum case, sensors with an output voltage of −10 V to +10 V can be read. The analog channels have 115 db of dynamic range that allows the resolution of signals that differ in amplitude by a ratio of over 560,000 to 1. In addition, the system allows CCLD (constant current line drive) sensors to be powered by the same signal reading line, eliminating the need for external signal conditioning. Microphone-type transducers are used together with Photon+. In this case the type-4188 microphone from Brüel and Kjær is used. The microphone has a high precision and sensitivity thanks to an integrated preamplifier. The sensor is capable of measuring signals between 20 Hz and 20,000 Hz and is powered by CCLD. The microphone sensitivity is 28.4 mV/Pa. Figure 4 shows the diagram of the sensors and acquisition systems used during the tests.

### 2.4. Auxiliary Pressure Line

The employed differential sensors are unidirectional and consist of two terminals, P_dat_ and P_ref._ When they measure a signal, they return a voltage proportional to the pressure difference dP = P_dat_ − P_ref_ if the pressure in the measurement is greater than the pressure in the reference. Similarly, if the pressure at the measurement terminal is lower than the pressure at the reference terminal (P_dat_ − P_ref_ < 0) the voltage saturates at 0 V, so it is not able to detect negative pressure differences. In order to allow the system to register pressure differences in both directions, an auxiliary pressure line connected to the reference terminal of the differential pressure sensors has been implemented.

**Figure 5:**
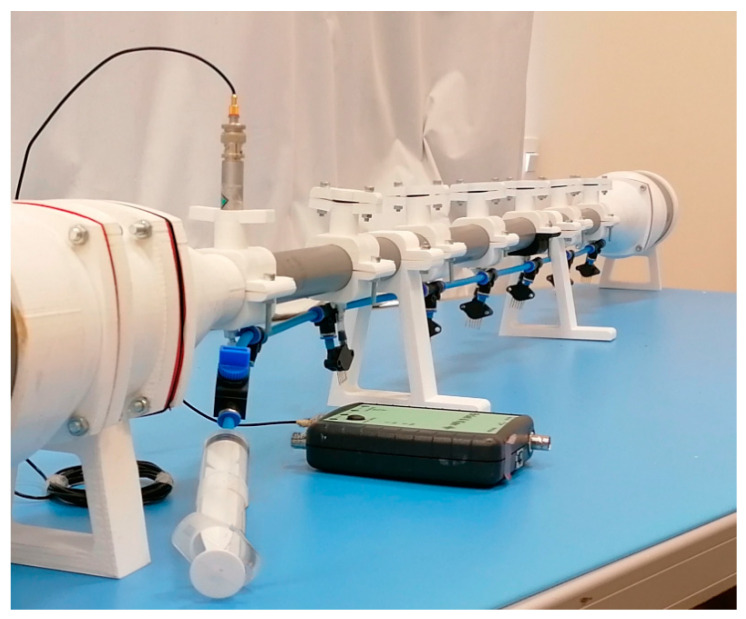
Pneumatic components used for the implementation of the auxiliary pressure line and its assembly on the acoustic bench.

Figure 5 shows the configuration of the pneumatic system implemented to the differential pressure sensors MPX5050DP. The reference terminal, P_ref_, is connected to an auxiliary pressure line that is sub-pressurized below atmospheric pressure. The pressure of the measurement terminal inside the resonator, P_dat_, is the atmospheric pressure, while the acoustic wave has not been generated by the acoustic driver. This raises the voltage recorded by the sensor in the absence of an acoustic wave to a value around the middle of the sensor’s full scale of 25 kPa. Thus, when the loudspeaker is activated, and condensations and rarefactions begin to occur in the air inside the resonator and the rarefactions of the fluid produce pressure drops, positive voltages will still be present.

The direct registration of the measurement of the differential sensor (dP = P_dat_ − P_ref_) with the system in the absence of an acoustic wave depends on what the reference pressure in the pneumatic circuit is reduced by moving the plunge of a syringe. In this way it is possible to detect the relative peak-to-peak pressure amplitude of the acoustic wave generated within the resonator by the speaker, from the difference between the maximum and minimum pressure of this wave.

In order to keep the pressure line common to all differential pressure sensors tight, pneumatic components are used such as the blue pneumatic line itself shown in Figure 5, and fittings are used to connect the pneumatic tube to the sensors. Once the desired reference pressure for testing has been established, a three-way shut-off valve with three positions is used to keep it constant during data acquisition: ON-OFF and ESC (discharge). This valve is used to close the circuit at the inlet, i.e., once the pressure has been introduced to the desired level, the valve closes, releasing the remaining air from the syringe into the environment, so that it can be handled without damaging the sensor. The measuring terminal of the differential sensor is installed directly at the bottom of the clamp shown in Figure 2 which is mounted on the resonator while the reference terminal of the sensor is connected directly to the auxiliary pressure line as shown in Figure 3. Consequently, the sensor works in its most efficient range, achieving high accuracy even at low signal-to-noise ratios, and at low cost.

## 3. Results

In order to assess the accuracy of the low-cost acquisition system implemented in the acoustic test bench, several tests were carried on using the Brüel and Kjaer professional measure system, described above, as baseline. Different pressure sine waves were generated through the bass speaker at different frequencies and amplitudes. The frequencies considered for the different experiments are 60, 80, 100, and 120 Hz. The low-amplitude and non-pressurized thermoacoustic engines can be designed to operate in linear mode, keeping the drive ratio (ratio of pressure amplitude to mean pressure) below 10%. At these pressures, which typically range from 3 to 8 kPa, both sensors and low-cost acquisition system are able to sample those signals accurately. However, measuring pressure waves’ amplitudes lower than 1 kPa become challenging for a low-cost system. In some cases, it is necessary to measure values of the pressure amplitude lower than 0.5 kPa, for example when an acoustic load is used to measure the dissipated power in the engine. To assess the accuracy of the low-cost pressure acquisition system in those situations, several experiments were carried out for sine wave amplitudes of 100 Pa and 225 Pa (200 Pa and 550 Pa peak-to-peak respectively).

### 3.1. Evaluation of Pressure Sensors

Initially, both absolute (MPX5100AP) and differential (MPX5050DP) pressure sensors were evaluated and compared to high sensitivity microphones. The signal provided by all the different sensors were registered by means of the Brüel and Kjaer acquisition board in combination with the RT Photon+ software. Different tests were carried on for sinusoidal signal of frequencies ranging from 60 to 120 Hz. Due to space considerations, in this communication only plots for 60 Hz sine wave test with peak-to-peak amplitudes of both 200 and 550 Pa are shown as examples of the results obtained. Following the methodology introduced by Alejandro et al. [6], the error introduced by the low-cost sensor is calculated as the root mean square (RMS) of the difference between the pressure values returned by the sensors and the baseline microphone (11). Where the RMS is defined as follows:

(11) $$Err=\sqrt{\frac{1}{N}\overset{N}{\underset{i=1}{\sum }}{\left(x_{i}-x0_{i}\right)}^{2}}$$

**Figure 6:**
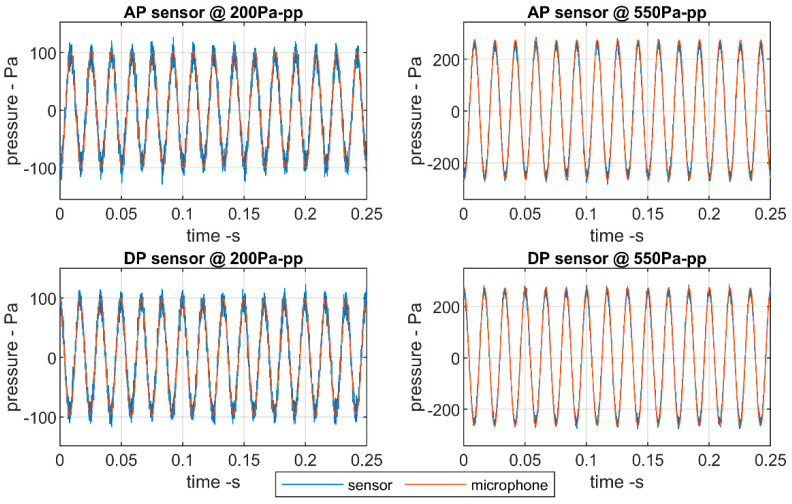
Detail of the comparisons between the sine wave signals of the low-cost sensors and the high-fidelity microphone.

Figure 6 presents the signals returned by the absolute (AP) and differential (DP) pressure sensors compared to the high-sensitivity Brüel and Kjaer microphone for sinusoidal sound waves of 60 Hz and peak-to-peak amplitudes of 200 Pa and 550 Pa. Although the pressure sensors return slightly noisier signals, both sensors are able to precisely follow the sinusoidal oscillations.

**Figure 7:**
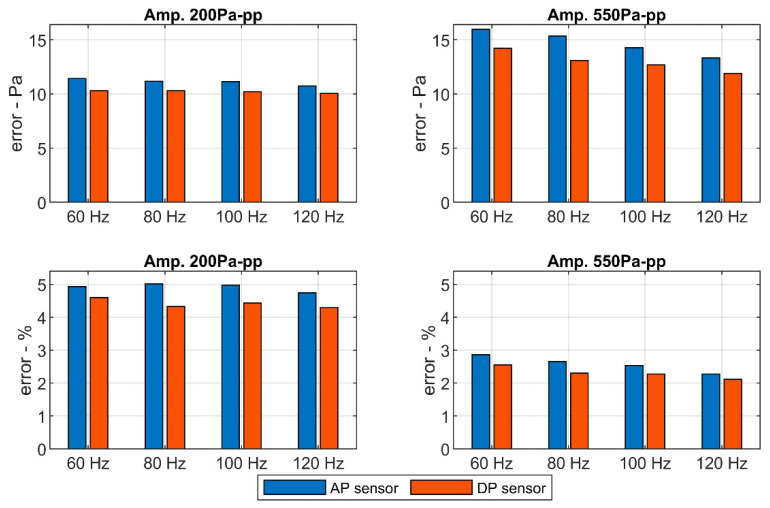
Error computed for the signals of the low-cost sensors with respect to the high-fidelity microphone.

For lower amplitudes, the noise ratio in the signal returned by the pressure sensors is higher. The results obtained for the differential pressure sensors do not differ much to those for the absolute pressure sensor. Although, the full scale of the differential pressure sensors is half of that of the absolute pressure ones. The errors introduced by the pressure sensors with respect to the baseline microphone are shown in Figure 7.

There exists a small difference between both sensors. The error introduced by the differential pressure sensor is slightly lower than the error introduced by the absolute pressure sensor. It may be due to the different full scales. However, the high-resolution of the Brüel and Kjaer system attenuates this difference. Errors are also lower for higher amplitudes being under 5% of the wave’s amplitude in any case.

### 3.2. Evaluation of the Low-Cost Acquisition System

One of the main goals of the present research is to develop a reliable low-cost acquisition system for acoustic purposes. The low-cost acquisition board chosen for the system is inspired in a previous work presented in [7]. It is based in an Arduino board; its sampling frequency reaches 3 kHz and its analog to digital converters have a resolution of 10 bits for a full scale of 5 V.

Two features of the low-cost acquisition board become of main relevance. On the one hand, the resolution of the ADC of the low-cost board is adequate to reproduce signals with those pressures that can be found in the acoustic network of a non-pressurized thermoacoustic engine (about 5000 Pa). However, when lower values of pressure are to be sampled (e.g., power measuring with an acoustic load), the limitation in resolution becomes more evident and the signal is sampled with bigger steps.

**Figure 8:**
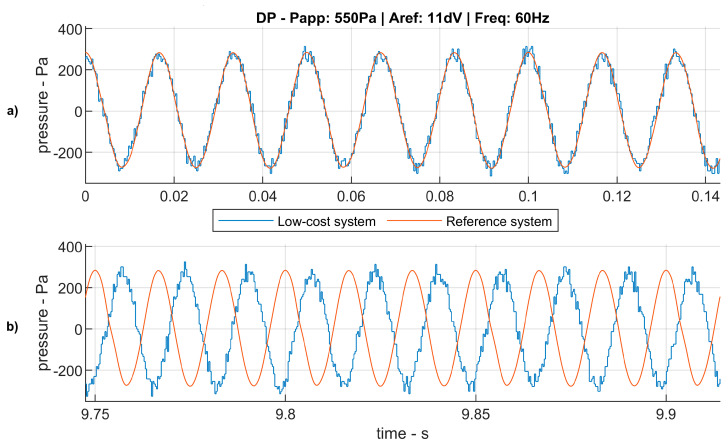
Detail of the time discrepancy between the low-cost and the high-fidelity acquisition systems. Both subfigures show the same signal at different time intervals. (**a**): Initial time interval of the signal. (**b**): Final time interval of the signal. After few seconds, the frequency discrepancy between the reference signal and the measure signal become visible.

On the other hand, the lower accuracy of the system’s timer produces time discrepancy between the reference signal (that acquired with the professional Brüel and Kjaer system) and the signal under study. The error in the time measured by both system is small compared to a wave’s period and mostly constant along the time. Nevertheless, the addition of small time errors after every sampling cycle, results in a clear mismatching of both signals after few seconds. In Figure 8, it can be appreciated how, in the first wave periods (Figure 8a), the sampled signals of the sound wave look to be in phase. However, after 10 s both signals are almost in phase opposition due to the cumulative time error.

In order to study both, the amplitude error and the time error introduced by the low-cost acquisition system, the signal sampled by this system has to be processed to match in time with the reference signal obtained with the Brüel and Kjaer system.

An algorithm which adjusts both signals is developed. This algorithm considers each maximum or minimum in the pressure wave as a unique event in time. Thus, the time array of the signal registered by the LC-DAQ (measure signal) can be scaled to be coincident in each event with the time array registered by the Brüel and Kjaer system (reference signal). The algorithm is structured in several stages:

(1) Samples number matching: The reference signal has a much higher sampling frequency than that of the measured signal. The number of samples of the reference signal is reduced in order to make it equal to that of the measured signal.
(2) Measured signal smoothing: A moving average filter is applied to the measured signal in order to smooth the signal noise and avoid mistakes in finding local extremes.
(3) Find local extremes: All the maximums and minimums in both measured and reference signals are located. The result of this operation are two arrays containing the time values registered for each extreme in both measure and reference signals.
(4) Time scaling: The corresponding extremes of the measured signal and of the reference signal are matched. Each semi-period starts with a maximum/minimum and ends with the next minimum/maximum. The following semi-period starts with the last minimum/maximum of the previous semi-period. The operations on each semi-period are recursively applied to all the semi-periods contained in the signals:
  (a) The process starts by assigning the time value of the first extreme of the reference signal to the first extreme of the measured signal.
  (b) Then, the time values of the measured signal are shifted according to the difference between the time value in the measured signal and the reference signal.
  (c) After shifting the time values of the measure signal, the difference between the measured and reference signals for the time value of the second extreme of the current semi-period is the time error that the LC-DAQ introduces. This error is registered for each semi-period of the wave.
  (d) Once the time error is found, the time value of the second extreme of the reference signal is assigned to the second extreme of the measure signal. The intermediate time values of the measure signal are assigned linearly. In this point, the current semi-period of both measured and reference signals are coincident in time.
(5) After the previous operations are carried out along the full sine wave, both reference and measured signals can be compared in amplitude. For this step, the original values of pressure of the measure signal are used, discarding the smoothed signal which was previously obtained to a better finding of the local extremes. The amplitude error introduced by the LC-DAQ is then computed as the RMS of the amplitude difference between the measured and reference signals for each time step.

Therefore, this algorithm returns, in the first place, a new time array for the LC-DAQ signal. Thus, the signals’ amplitudes can be properly compared. In the second place, it also returns an array containing the time differences detected between both signals for all the pressure wave’s periods sampled. These values allow to observe the trend in time deviation between the LC-DAQ and the Brüel and Kjaer systems. This is due to the difference accuracy of the clocks between the acquisition systems. The ATmega2560 microcontroller has a 16 MHz crystal oscillator with an accuracy of ±50 pulses per million. This causes an accumulation in the error as time passes during the measurement. The Photon+ system is designed for real time measurements of vibrations and acoustics. It has a high-performance DSP (digital signal processor). For the present investigation it is considered as an exact reference.

#### 3.2.1. Amplitude Error

**Figure 9:**
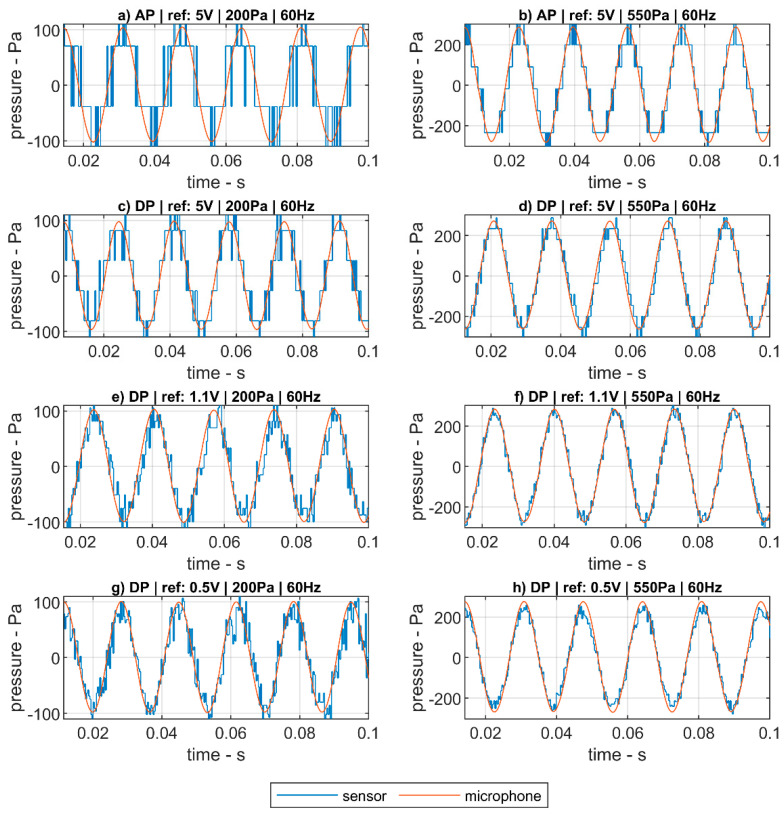
Detail of the improvement in sampling accuracy due to the different sensors’ arrangements: Absolute pressure sensor in (**a**,**b**); Differential pressure sensor with voltage reference of 5 V in (**c**,**d**); Differential pressure sensor with voltage reference of 1.1 V in (**e**,**f**); Differential pressure sensor with voltage reference of 0.5 V in (**g**,**h**). Column on the left represents the results for low pressure amplitudes: (**a**,**c**,**e**,**g**). Column on the right represents the results for high pressure amplitudes: (**b**,**d**,**f**,**h**).

As an example, in Figure 9 the results of measuring a 60 Hz sound wave with the pressure sensors sampled by the low-cost acquisition board, compared to the high-sensitivity microphones using the Brüel and Kjaer acquisition system, for four different configurations, are presented:

(i) Absolute pressure sensor (Figure 9a,b): The sine wave is registered with an absolute pressure sensor whose signal is sampled with the LC-DAQ system. The absolute pressure sensor is working close to the middle of it operational range, returning an average voltage of about 2.1 V and the ADC voltage reference of the acquisition system is set to the nominal value of 5 V.
(ii) Differential pressure sensor with voltage reference of 5 V (Figure 9c,d): The sine wave is registered with a differential pressure sensor whose signal is sampled with the LC-DAQ system. Taking advantage of the pressure line installed in the reference terminal of the sensor, the operational working point of the system is set in the high end of the sensor pressure range, returning voltage close to the 5 V limits. Thus, the ADC voltage reference of the acquisition system is set to the nominal value of 5 V.
(iii) Differential pressure sensor with voltage reference of 1.1 V (Figure 9e,f): The sine wave is registered with a differential pressure sensor whose signal is sampled with the LC-DAQ system. In this case, the operational working point of the sensor is set under the 1 V by reducing the negative pressure in the pressure line. Therefore, the ADC voltage reference of the acquisition system can be set to a value of 1.1 V and, thus, the relative resolution of the system is increased.
(iv) Differential pressure sensor with voltage reference of 0.5 V (Figure 9g,h): The sine wave is registered with a differential pressure sensor whose signal is sampled with the LC-DAQ system. Using the same approach than in the previous case, the ADC voltage reference of the acquisition system can be set to the value of 0.5 V.

A sinusoidal wave can be appreciated with the low-cost system looking at the signal in Figure 9b, with peak to peak amplitude of 550 Pa and for which the configuration (i) is used. However, due to its lower resolution, a steeped signal is obtained, for which the peak-to-peak amplitude is divided into six pressure levels. However, once the absolute pressure sensor is substituted by the differential pressure sensor with a reference voltage of 5 V, configuration (ii) in Figure 9d, the resolution of the signal is increased. A similar effect is achieved with configuration (iii) by further reducing the reference voltage to 1.1 V in Figure 9f. Nevertheless, for configuration (iv), where the differential sensor is working in its lower operational range below 0.5 V, the electrical noise overcomes the increase in resolution, as it can be appreciated in Figure 9h.

**Figure 10:**
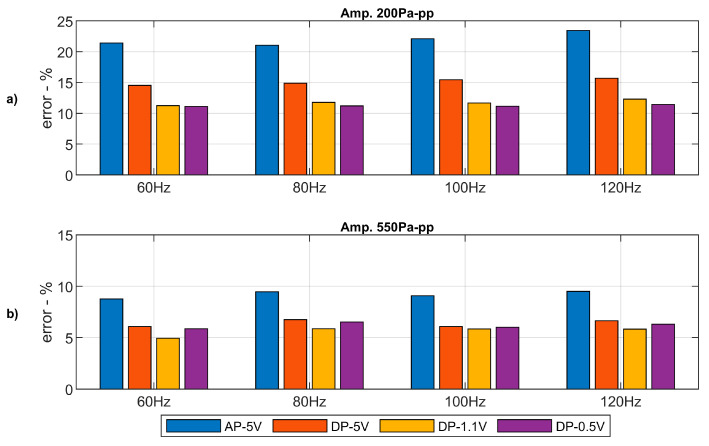
Relative amplitude error for the different sensors’ arrangements at different frequencies of the sound wave. (**a**): Lower amplitude signals. (**b**) Higher amplitude signals.

Similar results are observed for lower pressure amplitudes (Figure 9a,c,e,g). When the sine wave’s pressure amplitude is further reduced, the impact of lower ADC’s resolution in the low-cost system becomes more relevant and, thus, the improvement achieved by the different configurations. The results of the amplitude error obtained for the full frequency range with a pressure amplitude of 550 Pa are shown in Figure 10a. Similar results for an amplitude of 200 Pa are shown in Figure 10b.

The relative error introduced by the low-cost acquisition system is lower for higher amplitudes of the pressure wave. For the full frequency range under study, it can be observed how the differential pressure arrangement significantly increases the system accuracy for a reference voltage of 1.1 V. When the reference voltage is set to 0.5 V, the electrical noise becomes more relevant with respect to the voltage signal returned by the sensor. Thus, the system’s accuracy cannot be improved further in a significant manner.

Taking advantage of the sensors arrangement proposed in this work, for which differential sensors are used altogether with an adjustable reference pressure line and reducing the reference voltage up to 1.1 V, pressures waves with amplitude of 200 Pa can be sampled with an error of about 11%. Whilst an error lower than 6% is obtained when pressures waves with amplitude of 550 Pa are sampled. Considering that in the thermoacoustic circuit for which this acquisition system is developed the expected amplitudes are over 600 Pa, this last result can be accepted as the upper limit of the system error.

#### 3.2.2. Time Error

**Figure 11:**
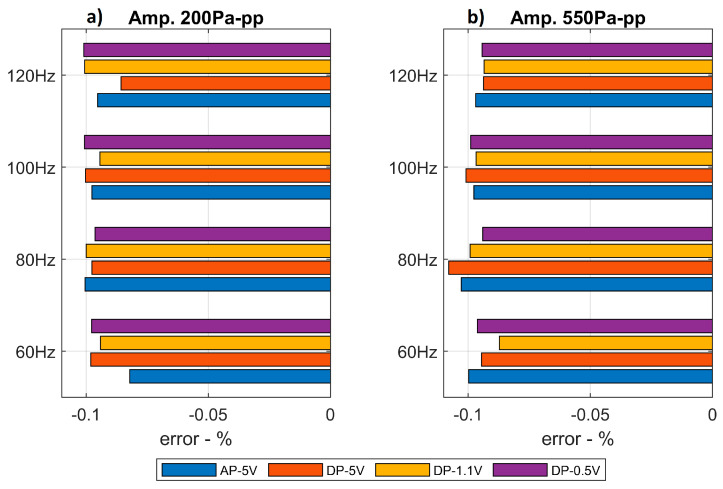
Relative time deviation for the different sensors’ arrangements at different frequencies of the sound wave. (**a**): Lower amplitude signals. (**b**) Higher amplitude signals.

As it is mentioned above, the LC-DAQ acquisition system’s timer is not as precise as that of the professional Brüel and Kjaer system. Consequently, an error is introduced in the sampling time. In order to study the time deviation between the low-cost system and the professional one, Figure 11 presents the average relative time error obtained in the different experiments with the different sensors arrangement and at different amplitudes and frequencies. Such a time error is computed as the difference between the final times registered by the two acquisition systems divided by the final time registered by the baseline Brüel and Kjaer system for each experiment and it is presented as a percentage.

It can be observed that the LC-DAQ system’s timer is slower in average and tends to introduce a delay of about 0.1%. This means that, for each second, the time registered by the LC-DAQ system is 1 ms shorter than that of the Brüel and Kjaer reference system. No relation has been found between the timer’s delay and the wave characteristics or the sensors arrangement implemented.

These results represent the global behavior of the low-cost system in terms of time measure. For a deeper understanding of such behavior, a statistical analysis has been carried out. Taking advantage of the algorithm developed to adjust both signals, the time discrepancy between them in each sine wave period is registered. Histograms of those deviations can be plotted, and those data can be adjusted to probability density functions. Considering that the sensors arrangement implemented does not affect the timer count, all the experiments for each frequency under study are grouped to obtain larger data samples.

**Figure 12:**
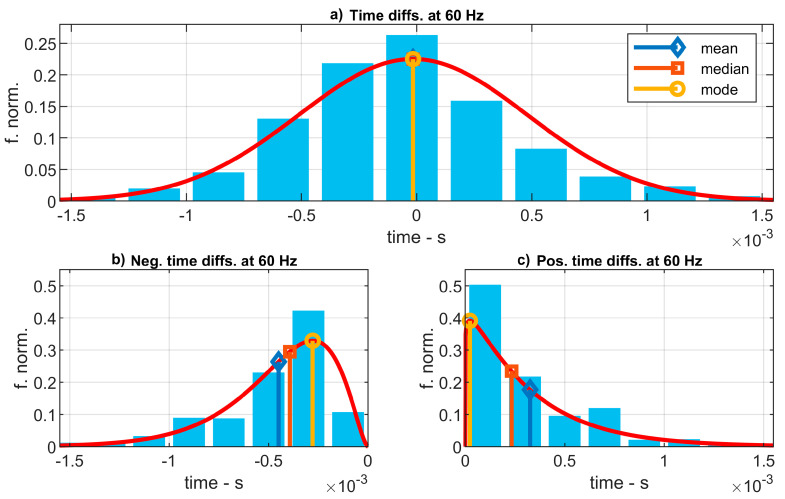
Histogram of the time errors registered for each sound wave’s period at a frequency of 60 Hz. (**a**): Errors are fitted to a normal distribution. (**b**,**c**): Positive and negative errors are separately fitted to gamma distributions.

As a first approach, Figure 12a shows the histogram of the time differences for the experiments at 60 Hz which has been fitted to a normal distribution. For this example, the distribution mean, median, and mode are about –15.6 µs. These results represent the average value of time delay that can be introduced by the low-cost system in the measure of two events separated in time by 1/60 s. This value is coherent with the results presented in Figure 11. It represents a −0.094% over a sine wave’s period of 1/60 s.

Nevertheless, these statistic values of time errors do not represent adequately the most probable time error that can appear in a time measure of the LC-DAQ system. Actually, most of the time errors registered were significantly larger, although, as those errors can be either positive or negative, the average value becomes much smaller in absolute value. To obtain a more precise representation of the time error introduced by the system, it is necessary to separate all the measures of time error between positive errors (time advance) and negative errors (time delay). Figure 12b,c present both positive and negative semi-histograms that have been fitted to gamma distributions, for which their means, medians, and modes are found independently for time advances and delays.

Following this model as a first approximation, the highest probability of a time error is a time advance of about 23.4 µs (see the mode in Figure 12c). Regarding to time delay, the most probable value is of −278.1 µs (mode in Figure 12b). Half of the positive time errors are below 232.2 µs (see the median in Figure 12c) and the average value of the time advances is 325.6 µs (see the mean in in Figure 12c). Half of the negative time errors are over 393.5 µs (median in Figure 12b) and the average value of the time delays is −449.1 µs (mean in Figure 12b). These results are coherent with those presented in Figure 11, for which there exists a general trend of the system to introduce a delay.

**Figure 13:**
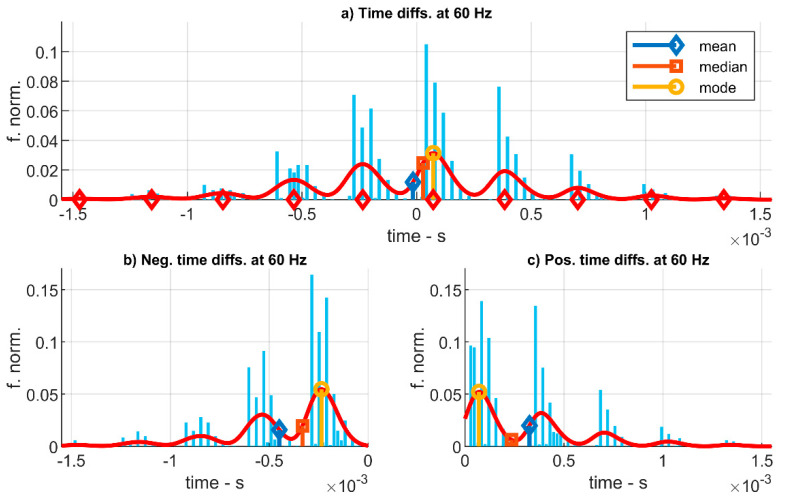
High-resolution histogram of the time errors registered for each sound wave’s period at a frequency of 60 Hz. (**a**): Errors are fitted to a kernel distribution. (**b**,**c**): Positive and negative errors are separately fitted to kernel distributions.

Looking for a more precise description, different histograms of higher resolution (higher number of bins) were performed for the full frequency range. Figure 13a shows the high-resolution histogram of the time differences for the experiments at 60 Hz. As the resolution is increased, a remarkable effect appears in the histogram: the time errors look to be grouped around certain values. In order to fit these data to a probability density function that accurately describes the behavior of the time error in our system, a kernel distribution is used. This behavior occurs in all the different experiments, with the different sensors’ arrangement and at different frequencies and amplitudes. Furthermore, if the all the experiments’ data are considered altogether, the resultant distribution present the same effect.

**Figure 14:**
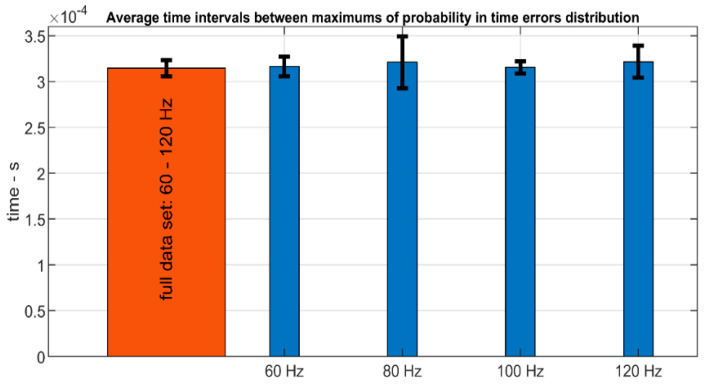
Time distance between maximums of probability for time errors distributions.

When the time distances between the peaks of maximum probability of the kernel distribution are analyzed, it is found that these distances are always close to 314 µs in all the different experiments. Figure 14 shows this time distance for the full frequency range. In orange, it is represented this value when all the experiments’ data are grouped to be fitted as a unique distribution. In blue, this value is represented for the different distributions obtained with the data of the experiments carried out at different frequencies. The standard deviations for each distribution are shown with black forks.

At this point, we have not found what causes this effect. The time resolution obtained with the LC-DAQ (which is based on an ATmega2560 microprocessor) is 1 µs. The value of the time registered by the system is directly taken from the system’s clock in each sample. Thus, neither the communication protocol between the LC-DAQ and the computer nor the delay introduced by firmware’s operations can modify this time value. This effect might be due to some process related with the system’s timer engineering. However, deeper research is needed in order to get a clearer view of this effect and to be able to obtain more solid conclusions. Nevertheless, this is beyond the scope of this research, which is to introduce a low-cost acquisition system able to register the pressure waves in thermoacoustic circuits and to characterize its accuracy.

**Figure 15:**
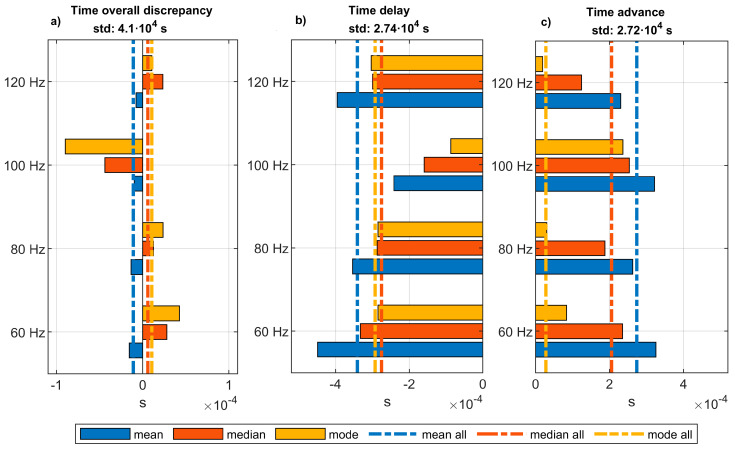
Mean, median, and mode of the time errors. Bar diagrams represent these statistics for the probability density functions obtained by fitting to kernel distributions the data from the experiments at different frequencies. Dashed lines represent the values of the statistics for the global distribution including the data coming from all the experiments at the different frequencies. (**a**): Presents the overall discrepancy in time, for which both positive and negative time differences are considered. (**b**): Presents the negative time differences (time delay). (**c**): Presents the positive time differences (time advance).

In order to characterize the time error introduced by the low-cost system, the time errors obtained for the different experiments at different frequencies are fitted to kernel distributions to obtain the mean, the median and the mode of each distribution. Figure 15a shows these values when both positive and negative time deviation are considered in one distribution. Figure 15b represents similar statistics for the negative time errors and Figure 15c, for the positive time errors. In all the three figures, dotted lines are superposed to present the mean, the median and the mode of the time error distribution of the whole dataset. This is, when all the data from the different experiments at different frequencies are grouped in one single kernel distribution. The standard deviations of these global distributions are also shown in the title of each sub-figure.

The probability density function obtained by fitting the data collected from all the experiment and for all the frequencies under study can be used to characterize the expected time error introduced by the low-cost acquisition system.

On the one hand, if both positive and negative time errors are fitted to a kernel distribution (Figure 15a), the mean of these distributions represents, for each frequency, the expected value of the time delay introduced by the LC-DAQ for each wave’s period in an extended time sample. Whilst the mode represents the most probable value of time error that can be found in a random measure of two events separated in time by periods of 1/60 s, 1/80 s, 1/100 s, and 1/120 s, respectively. On the other hand, considering positive and negative time errors separately allows obtaining a wider view of the time error behavior.

**Figure 16:**
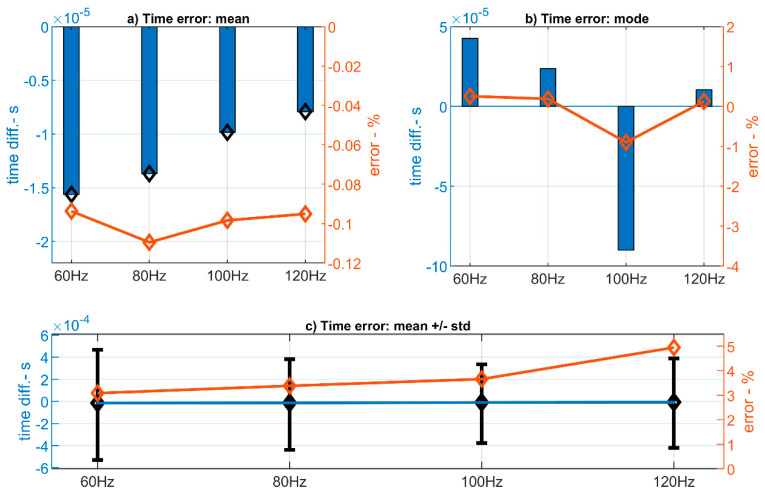
Estimation of time error through (**a**) the mean, (**b**) the mode, and (**c**) the addition of the mean and the standard deviation.

It can be observed that the mean value of time errors of the distributions considering both positive and negative values, follows a relation with the frequency of the signal. If these values are normalized with the wave’s period, a relative error can be obtained. Figure 16a presents the mean values of the time overall discrepancy for each frequency under study in a blue bar diagram. The overlying orange line represents these values multiplied by the wave’s frequency in each case and multiply by 100. This is the percentage of time deviation over the wave’s period, which stays around 0.1%.

If similar analysis is performed over the distributions’ modes (as the most probable value of error returned by the system) the relative error is increased as it is shown in Figure 16b. In this case the relative error reaches values up to 1% in the case of the 100 Hz experiments.

However, both mean and mode do not adequately represent the time error that could be expected in a single measure of time between two events. A more realistic value of error can be obtained by adding the standard deviation of the time error to the mean value. Figure 16c represents the mean of the time errors (blue line) along with the addition of the standard deviation represented by black forks. The relative error is computed by taking the largest value of the forks’ tips for each frequency and multiplying it by the frequency itself. It is plotted in an orange solid line. As expected, the relative error become higher in this case ranging from 3% at 60 Hz up to 5% at 120 Hz. It can be observed that, for higher frequencies (shorter time intervals between samples) the relative error increases.

## 4. Discussion and Conclusions

The accurate and low-cost measurement of dynamic pressure and its stability remains an important issue for thermoacoustic experiments. In the present research, a low-cost acquisition system has been introduced to quantify the levels of accuracy that can be achieved by differential pressure sensors and a settable reference pressure arrangement that allows significantly increasing the output signal resolution. The low-cost system has been tested on an acoustic test bench replicating working conditions in thermoacoustic machines. This is particularly valuable if the measured dynamic pressure is expected to compare reliably with theoretical predictions.

The system’s accuracy has been satisfactorily assessed for the conditions expected in thermoacoustic experiments in terms of frequency and dynamic pressure.

Initially, the low-cost pressure sensors were evaluated by comparison with professional microphones, registering all signals with a high-fidelity acquisition board. The relative error introduced by the low-cost sensors was found under 5% in the most demanding case for signals of 200 Pa peak-to-peak amplitude and under 3% for signals of 550 Pa peak-to-peak amplitude.

Secondly, the low-cost acquisition board has been evaluated in terms of pressure resolution and timer accuracy. By setting the system’s reference pressure at low values and reducing the ADC voltage reference of the low-cost acquisition board, the error of the system could be significantly reduced with respect to the initial error produced by the system when absolute pressure sensors were used. With the final configuration, the total error of the system for amplitudes of 200 Pa peak-to-peak, is under 12%. Nevertheless, for amplitudes of 550 Pa peak-to-peak, the relative error is under 6%. Considering that, in the thermoacoustic experiments for which this system was developed, the minimum pressure amplitude obtained was always over 550 Pa peak-to-peak, this error value can be taken as the upper limit value of the whole system’s error. The lower limit value is imposed by the error of the pressure sensor itself, which cannot be reduced even for larger wave’s amplitudes. Thus, it can be said that the pressure’s error introduced by the low-cost system is between 3% and 6% for the thermoacoustic pressures range for which it has been designed. This error is increased when sine waves of amplitude lower than 225 Pa are measured, reaching a value of 12% in the case of wave’s amplitudes of 100 Pa.

Regarding to time accuracy, two factors were studied: On the one hand, the overall error in the time span of a long acquisition process of several seconds while, on the other hand, the time discrepancy between the low-cost and the baseline system in the measure of two events close in time, for instance to peaks of the same sound wave.

In the first case, it is found that the low-cost system introduces a short delay of about 0.1%. This means that the time registered by the low-cost system is 1 ms shorter for each second than that of the baseline system.

In the second case, determining the error produced by the low-cost system in the measure of two events close in time is not direct. The time error does not follow a normal distribution as it could be expected. It does not even follow gamma distributions when positive and negative time discrepancies are considered separately. It has been found that the time errors, in this case, tend to group around certain values separated by 314 µs ones from the others. This happens for the different amplitudes, frequencies, and arrangements under study. That being so, the time discrepancy was modelled as kernel distributions for the different frequencies to obtain the expected mean, median, mode, and standard deviation.

When the mean value is considered for each frequency, the relative error obtained is close to 0.1% in the full frequency range. This is coherent with the results obtained for long time measures. However, when the mode value of the time discrepancy is considered as the most probable value of the error, the relative error rises to 1%.

If the addition of the mean and the standard deviation is taken as the approximated value of the expected time error for the measure of two events close in time, the relative error is increased with the frequency. As closer are the two events in time, the time error represents a bigger percentage over the sine wave’s period. In the present research, the highest frequency studied was 120 Hz, for this case the relative error reaches 5%. The lowest frequency was 60 Hz, being the relative error for this frequency 3%. Finally, the lower limit of this relative error would be defined by that obtained for measures of a long-time span, which was found as 0.1%.

The acoustic sampling system developed in this research has proven to be an adequate low-cost system for those thermoacoustic circuits for which has been developed and for any other systems working in similar conditions, e.g., a frequency’s range of 0–120 Hz, and for pressures above 225 Pa. For these conditions, both pressure and time errors introduced by the whole system are below 6% in the worst case. Although its accuracy increases for lower frequencies and higher pressures.

The unexpected behavior of time error found in this research leads to questions about the internal timer system of development boards such as this used in the present communication. Further research will be carried out to study this effect on different microcontroller and board arrangements in order to be able to make better predictions and improve the accuracy of low-cost systems based on these devices.

Finally, the acoustic test bench presented becomes a useful tool for our future research, which allows generating and analyzing pressure signals, emulating those in thermoacoustic engines, under controlled conditions.
